# Italian Consensus and Review in Lower Face Mimetic Muscle Treatment with Botulinum Toxin-A

**DOI:** 10.3390/toxins18080332

**Published:** 2026-07-31

**Authors:** Salvatore Fundarò, Paola Molinari, Giovanni Brunelli, Maria Cazzulani, Guido Dalla Costa, Roberto Dell’Avanzato, Roberta D’Emilio, Carlo Di Gregorio, Antonella Franceschelli, Monica Renga, Giovanni Salti, Adriano Santorelli, Ugo Simone Urso, Ilaria Zollino

**Affiliations:** 1 Qute Clinic, Via Zucconi 92, 41126 Modena, Italy; 2 Private Practice, Via Pietro Giardini 45, 41124 Modena, Italy; paola.molinari2002@gmail.com; 3 Private Practice, Via Guido Zadei, 64, 25123 Brescia, Italy; giovannibrunelli@gmail.com; 4 Master of Aesthetic Medicine of the Face, Unicamillus International Medical University, 00131 Rome, Italy; maria.cazzulani@hotmail.it (M.C.); afranceschelli@me.com (A.F.); 5 Private Practice Clinica Cittàgiardino, Via Francesco Piccoli 6, 35123 Padova, Italy; guidodallacosta@gmail.com; 6 Rigenera Care, Via Carlo Marenco 32, 10126 Torino, Italy; dellavanzato@hotmail.it; 7 Private Practice, Via Filippo Antonio Gualterio, 127, 00139 Roma, Italy; demilioroberta@gmail.com; 8 Plastic Reconstructive Aesthetic Surgery Private Practice, Via Roma, 325, 90133 Palermo, Italy; cdigregorio@hotmail.com; 9 Private Practice, Via della Moscova, 40/1, 20121 Milano, Italy; monica.renga@gmail.com; 10 Private Practice, Via Claudio Monteverdi, 2, 50144 Firenze, Italy; giovannisalti@gmail.com; 11 Private Practice, Via Raffaele Morghen, 88, 80129 Napoli, Italy; dott.adrianosantorelli@gmail.com; 12 Private Practice, Via Roma, 2, 40069 Zola Predosa, Italy; urso@studiomedico109.it; 13 Private Practice, Via Marcello Tassini, 6, 44123 Ferrara, Italy; ila.zollino@gmail.com

**Keywords:** botulinum toxin, consensus, aesthetic, lower face, mentalis muscle, depressor anguli oris, platysma

## Abstract

Background: Treatment of the lower face with botulinum toxin type A (BoNT-A) is particularly challenging because of the important functional roles of the involved muscles, which increases the risk of adverse events and complications. Methods: To address this issue, a panel of 14 experts conducted a three-round consensus study, including digital surveys and a formal meeting, on the aesthetic treatment of the mentalis (MT), depressor anguli oris (DAO), and platysma (PL) muscles using BoNT-A. The study was complemented by a literature review of 16 international consensus papers published between 2004 and 2024. Results: The primary indications for MT treatment that achieved strong consensus were “peau d’orange” (“golf ball”) chin appearance and hyperactivity. The Italian panel recommended a mean total dose of 8 U (distributed bilaterally), which was higher than the international mean dose of 6.5 U. The preferred injection technique consisted of a combination of intramuscular and intradermal injections. For the DAO, the strongest indication was correction of downturned oral commissures. Both the Italian panel and the international consensus reported a mean total dose of 5.6 U. Most experts (92.8%) preferred intramuscular injections, typically administered at a single injection point lateral to the marionette line in order to remain within a safe treatment zone and avoid diffusion to the depressor labii inferioris muscle. Indications for PL treatment included platysmal bands, jawline recontouring, and improvement of skin texture. The techniques discussed included treatment of both platysmal bands and the jawline. The consensus panel recommended a significantly higher mean total dose (48.4 U) than the international average (38.2 U). The paper also highlights the growing importance of ultrasound imaging as a tool for identifying individual anatomical variations in the lower facial musculature. Conclusions: As the demand for lower face rejuvenation continues to increase, standardized injection techniques and the integration of ultrasound imaging are essential for minimizing functional complications and optimizing aesthetic outcomes.

## 1. Introduction

Facial expression muscles differ from skeletal muscles because they originate from bone and insert into the skin via the superficial musculoaponeurotic system (SMAS) [1]. In contrast, most other muscles of the body have both bony origins and insertions. This unique anatomical feature enables facial muscles to generate the skin movements responsible for facial expressions, thereby playing an essential role in interpersonal emotional communication [2]. Contraction of the underlying muscles causes skin movement and folding, leading to the formation of dynamic wrinkles that are usually oriented perpendicular to the muscle fibers [3]. Over time, this process may result in dermal atrophy and skin pleating, contributing to the appearance of facial aging and creating an angry, sad, or tired expression.

The treatment of dynamic wrinkles with botulinum toxin type A (BoNT-A) has been performed for many years and is widely used for muscles of the upper third of the face, including the frontalis, corrugator, and orbicularis oculi muscles. More recently, treatment has also been extended to muscles of the lower face, such as the mentalis (MT), depressor anguli oris (DAO), and platysma (PL).

Facial muscles not only serve an expressive function, but also play an active role in the function of structures such as the nose, eyelids, mouth, and other facial regions. Compared with their mimetic role, functional activity is more prominent in the perioral muscle group than in the periorbital region. Consequently, the risk of complications resulting from excessive neuromodulation or inadvertent involvement of non-target muscles is greater in the perioral area. Recently, ultrasound imaging has been increasingly used to evaluate muscle anatomy, identify individual anatomical variations, and facilitate safer and more effective BoNT-A treatments. The muscles of the inferior perioral group (MT, DAO, and PL) are frequently treated in daily clinical practice with favorable outcomes; however, they are also associated with a significant rate of complications. For this reason, the use of ultrasound guidance for the treatment of these muscles is particularly recommended and may eventually become standard practice. This article aims to review the anatomy, the literature regarding BoNT-A injection techniques and the use of ultrasound in the assessment and treatment of the MT, DAO, and PL muscles. To further define treatment approaches for these muscles, we conducted an Italian consensus involving 14 expert injectors and compared the consensus outcomes with the available literature. The review of anatomy, injection techniques, and ultrasound imaging was an integral part of the discussion that led to the consensus, and therefore we believe it is appropriate to report it as a fundamental introduction to the consensus.

**Anatomy of the mentalis muscle.**

**Figure 1:**
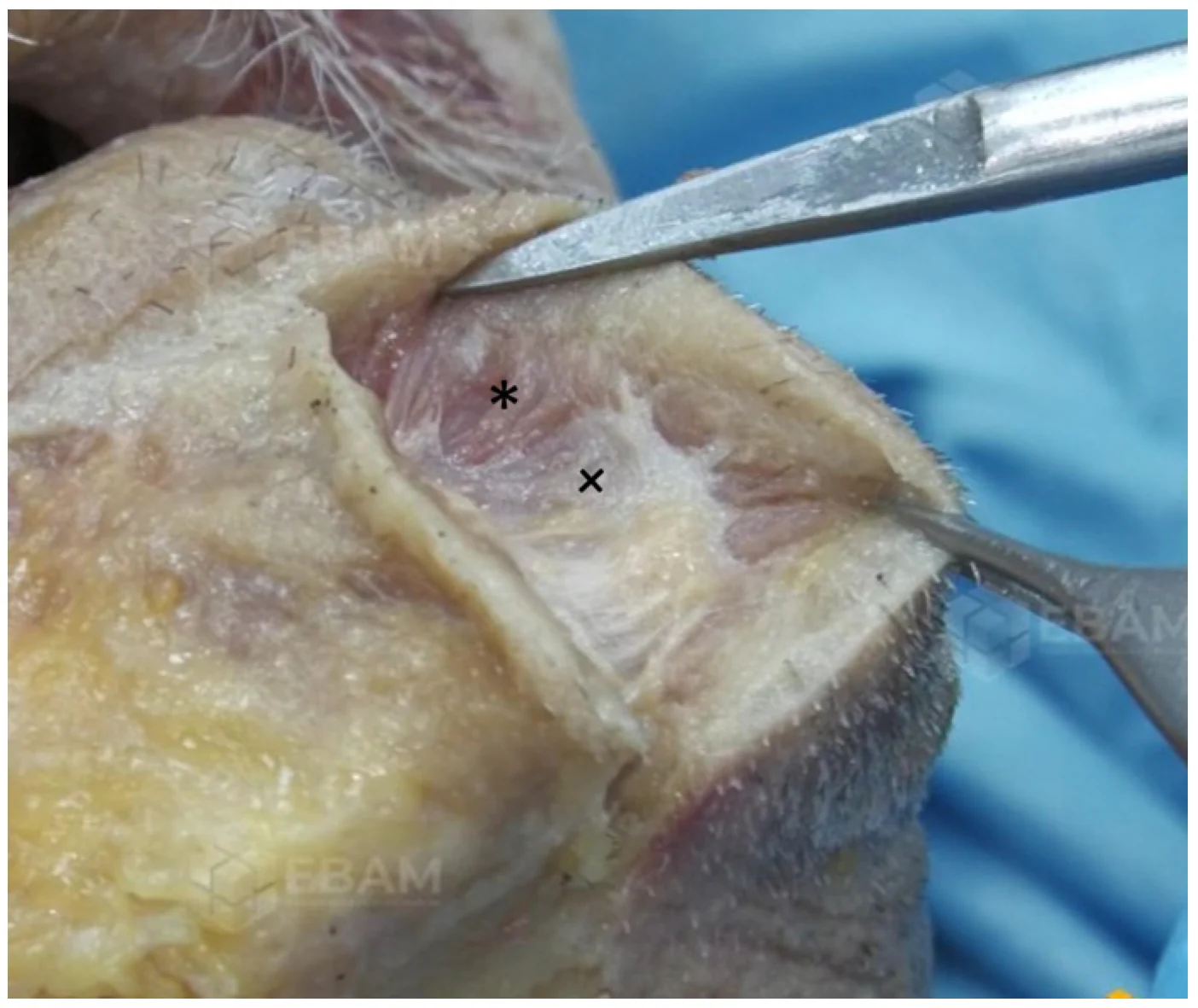
Anatomy of the mentalis muscle: the upper fibres are short and run horizontally (star), the lower fibres are long and descend inferomedially or vertically (cross). Its fibres originate from the alveolar bone of the anterior mandible and insert into the dermal layer of the chin. The connective–adipose tissue between the two mentalis muscles has been removed to expose the muscular fibres.

The mentalis muscle (MT) is the only elevator of the lower lip and chin and provides the primary vertical support for the lower lip [4]. Its fibers originate from the alveolar bone of the anterior mandible, approximately 2 cm inferior to the intercheilion line and 0.8 cm lateral to the midline of the chin. The upper fibers are short run horizontally and intermingle superiorly with the inferior margin of the orbicularis oris (OO) muscle, whereas the lower fibers are longer and descend inferomedially or vertically. The medial fibers descend anteromedially in a dome-shaped configuration, while the lateral fibers descend obliquely and attach to the ipsilateral skin partially intermingling with the depressor labii inferioris (DLI) muscle. The MT inserts into the dermal layer of the chin at a more medial position than its origin. The two mentalis muscles are usually separated at their origins by connective–adipose tissue (Figure 1) [5,6,7].

The anatomical relationship of MT with OO and DLI may lead, due to the diffusion of BoNT to these muscles, to asymmetric expression changes in the lower face, and complications such as dysfunctional mouth action, ptosis of the lower lip, as well as asymmetrical smiling.

The motor endplates of MT could be found in both superior and inferior muscle portions, with a predominance of the superior location. The superior endplates were concentrated in a relatively delimited area, meanwhile the inferior endplates were more widely distributed in the muscle’s caudal portion. The motor-endplates mainly lie approximately between 25 and 50% of the distance from the inferior border of the lower lip vermilion to the median inferior border of the mandibula [8].

**Injection techniques of mentalis muscle.**

Disparate injection techniques for MT treatment with BoNT-A have been described, all trying to reduce the related complications and optimise the results. Le Louran [9] suggested superficial injection into the upper part of the muscle to creates a chin advancement with softening of the skin. Carruthers and Carruthers [10] suggested a single distal-most point from the OO to the MT as an injection point. Similarly Beer, Braz, D’Emilio, Raspaldo [11,12,13,14] suggest one single intramuscular injection point in each MT. Other authors [15,16] suggest the injection in one single point on the midline which may be combined with the two bilateral points technique. Some consensus papers describe intramuscular injection in one, two or four points per side [17,18]. Based on a literature review, the units injected per side vary from 1.5 U to 5 U [19,20,21,22,23,24,25,26,27].

**Ultrasound imaging of mentalis muscle.**

The ultrasonographic imaging of the MT muscle provides detailed information about its internal structure, morphology, location, and dimensions, which is crucial for safe and effective botulinum neurotoxin (BoNT) injections [28].

Choi et al. [29] determined by ultrasonographic study and cadaver dissection the anatomical morphology, location, and depth of the MT muscle to provide crucial information for clinical procedures, particularly botulinum toxin injections for lower face rejuvenation. In this article, the MT muscle was classified into two main types based on its shape from cadaveric dissections: type A and type B. The type A (dome-shaped): this was the predominant type, accounting for 86.4% (38 out of 44 cases) of the specimens. This type of MT muscle was further divided into two subtypes: Type A-1 (named “unified dome”), where the two bilateral MT muscles merged (47.7%, 21 cases), and Type A-2 (named “twin peaks”), where the two MT muscles were separated (38.6%, 17 cases) with space in between, often containing connective tissue and some fat. The type B (flat horizon) was characterised by only a few muscle fibers, making them appear quite thin and less three-dimensional, typically separated and trapezoidal in shape (13.6%) [29,30].

**Figure 2:**
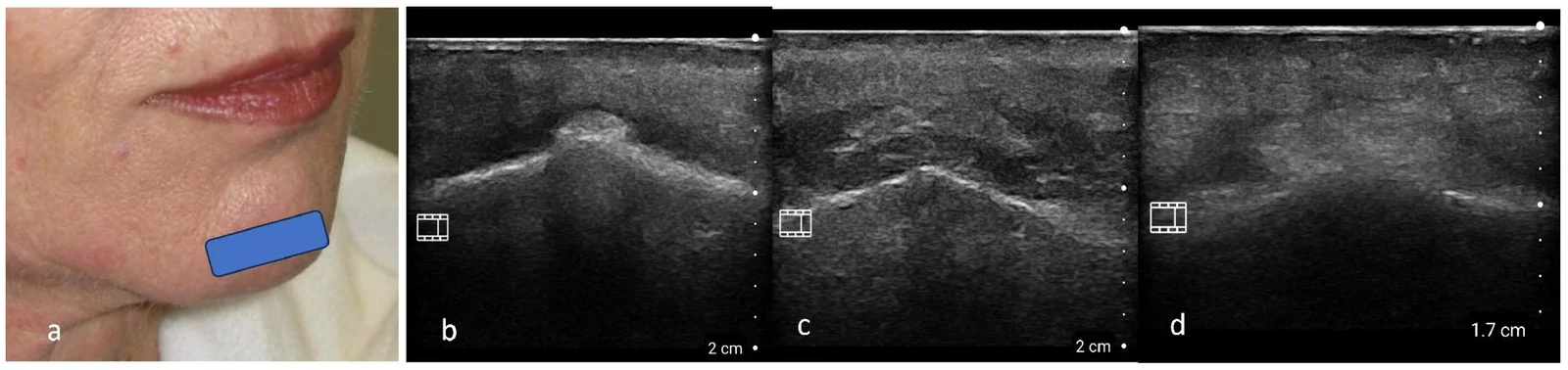
Ultrasonographic images of mentalis muscle: (**a**) The blue rectangle represents the position of the 20 MHz probe positioned horizontally on the chin. Three different types of images may be identified. (**b**) Type A-1 appears as a dome-shaped hypoechoic area that merges at a more superficial part of the chin. Between the two mentalis muscles, the hyperechoic connective-adipose tissue is well evident. (**c**) Type A-2 is dome-shaped, but the muscle fibres on each side are separate. (**d**) Type B: two separated hypoechoic areas are located lateral to the midline and are completely separated by connective fatty tissue.

These three morphological types (A-1, A-2, B) can also be clearly distinguished using a 20 MHz probe positioned horizontally to the chin, 5 mm above the pogonion (Figure 2a). Type A-1 appears as a dome shaped hypoechoic area that merge at a more superficial part of the chin (Figure 2b). Type A-2 is dome shaped but the muscle fibers on each side are separate (Figure 2c). In type B MT muscle, two separated hypoechoic areas are located laterally the midline, and are completely separated by connective fatty tissue (Figure 2d).

Choi et al. [29] determined by ultrasonography the MT muscle measures and anatomical extension. It is present mostly in the area 5–10 mm from the facial midsagittal line and 20–30 mm from a horizontal line connecting the mouth corners. The MT muscle was found to be, on average, 6.7 ± 1.4 mm below the skin surface and was 1.1 ± 1.0 mm from bone surface. This new information about the morphology, location, and depth of the MT muscle is considered useful for identifying the most effective and safe BoNT-A injection points and depths during esthetic procedures for weakened facial rhytides on the lower face. They suggest that injecting BoNT-A at a depth of about 9 mm will accurately target the muscle layer in most cases. A single injection into an appropriate location and depth could be sufficient to paralyze the entire muscle. However, due to toxin spread depending on dose and volume, multiple point injections with distributed total dose are known to help contain the effect and can be safer and more effective, especially with the aid of ultrasonographic evaluation. Nazari [30] identifies by ultrasonography superficial and deep fibers which have different actions: the superficial attach just under the derm and they compress certain segments of the dermis, causing small dimples or depressions in the skin (“Cobblestone Chin”), whereas deep fibers elevate the chin’s soft tissue, enhance the mental crease, and protrude the lower lip. The author, combining the Choi classification and evaluation of deep or superficial fiber activity, suggests three injection techniques. In type A1, two intradermal injections at pogonion and gnathion (1 U) and intramuscular injection (2–3 U) 5 mm lateral to pogonion. In type A2, one deep injection (2–3 U) and one superficial (1 U) is suggested. In type B, injection 5 mm lateral to the pogonion into the deep fibers (3 U) is recommeneded. Yi et al. propose, based on ultrasonographic assessment of MT anatomy, a similar technique: one intramuscular 3 U injection 0.5 cm lateral to the pogonion, followed by a superficial injection of 1 U per side at the same point performed during needle extraction [28].

**Anatomy of the depressor anguli oris muscle.**

**Figure 3:**
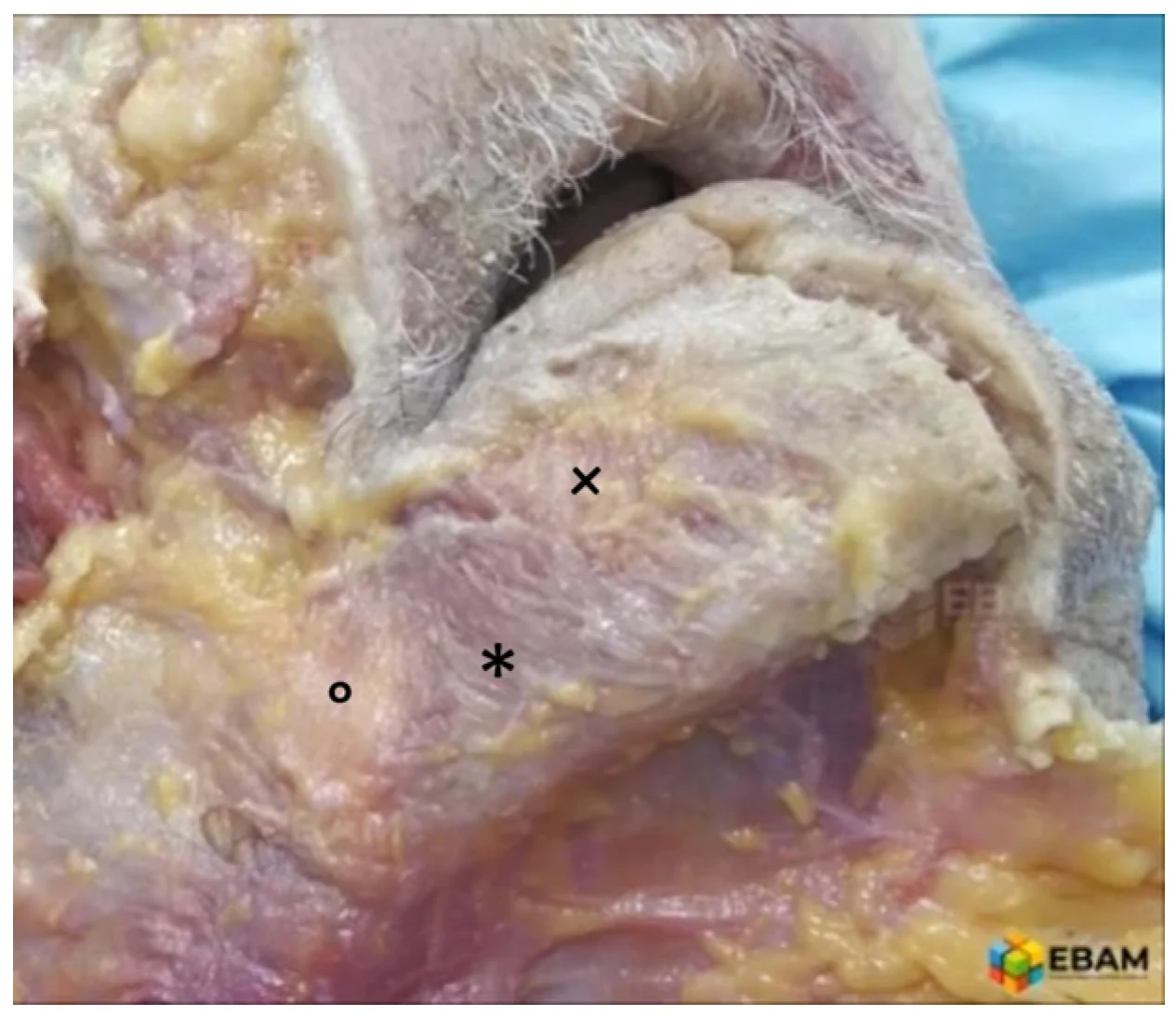
Anatomy of depressor anguli oris: DAO (star) is a triangle-shaped muscle located under the subcutaneous layer of the inferior and lateral perioral region. Its fibres originate just lateral to the mentum from the linea obliqua mandibulae, run upward, converge in a narrow fasciculus at the oral commissure and insert into the modiolus. The DAO and DLI (cross) partially overlap inferiorly; the platysma fibres (circle) reach the lateral border of the DAO and continue to run under the DAO.

The depressor anguli oris (DAO) is a perioral triangle-shaped muscle located on the most superficial layer of the inferior and lateral perioral region. Its fibers origine just lateral to the mentum from the linea obliqua mandibulae, run upward above and laterally the depressor labii inferioris (DLI). They travel upward and converge in a narrow fasciculus at the oral commissure (Figure 3).

Some of them insert directly into the dermis of the skin. In contrast, others insert into the modiolus and interdigitate with muscle fibers of the risorius, OO, and PL [31,32]. DAO inserts at the modiolus, a thick fibro-muscular band formed by the converging of the zygomaticus major, DAO, levator anguli oris, buccinators, OO, and risorius muscles [33]. The modiolus in Asians is positioned more caudally than the intercheilon line compared to Caucasians. Hyperactivity of the DAO muscle pulls the modiolus downward, leading to drooping of the mouth corner, which can result in a depressed and gloomy expression [34], and, in combination with ageing process, create the appearance of the labiomandibular fold [35]. The DAO and DLI partially overlap inferiorly, with the DLI lying beneath the DAO and DAO fibers may be connected and intermingled with fibers of MT and DLI muscles. The partial overlap and fibers blending may cause dysfunctional movement of the lower lip and an asymmetric smile due to unintentional neuromodulation of the DLI and MT muscles [36].

The motor endplates distribution in the DAO is generally characterized by a dominant motor zone, often combined with a second smaller motor zone. The DAO motor endplates are concentrated in a band-shaped area extended in the mediolateral direction, which is located slightly inferior to half the distance between the modiolus and the mandibula’s lower border [8].

**Injection technique of the depressor anguli oris.**

Several injection techniques for DAO treatment with BoNT-A have been described; mainly, they differ in number, location, injection level, and units used. Le Louarn suggests that the BoNT-A should be injected as close as possible to the muscle’s motor endplates. He describes injecting 1–4 units into the subcutaneous fat in front of the DAO at its mid-muscle height in the marionette fold. To effectively block the maximum contraction force of DAO, an injection of 3 to 4 units is recommended. Choi et al. [34] advise using the modiolus as an anatomical landmark and injecting BoNT-A into the DAO muscle within a triangular area located less than 45 degrees laterally and less than 30 degrees medially to the vertical line passing through the modiolus. The same author [37], by 3D scanning and cadaveric dissections, maps the anatomical overlapping of DAO and DLI. He identified a specific intersection point between the mid-pupillary line and a horizontal line from the pogonion to otobasion inferius. This “DAO point” assures a safe and accurate injection protocol that can minimize unintended side effects. A similar injection technique is proposed by Yi et al.: the injection site is along the mid-pupillary line at 15 mm from the lower margin of the mandible for Caucasians and at 12 mm along the lower margin of the mandible for Asians. Intradermally or subdermally one point injection is proposed with 2–3 units (U) at each side [38]. A single site of injection is also used by Fabi et al. [39] in a split-face comparison of onabotulinumtoxinA and abobotulinumtoxinA. Injection sites are immediately above the angle of the mandible and 1 cm lateral to the lateral oral commissure. Four U of onabotulinumtoxinA in one depressor anguli oris and ten U of abobotulinumtoxinA in the contralateral muscle. Other authors [40] inject 4 U in the upper 1/3 of the DAO. According to Qian et al. [41], the injection dose is determined based on the severity of the drooping mouth corner. Usually, 2–4 U per site are used, distributed across 1–3 superficial injection points, situated 8–10 mm outside and 8–15 mm below the mouth corner. Moradi and Shirazi [32] propose a three injection points technique: injections are administered along a tangential line from the oral commissure to the mandibular edge, typically along the labiomandibular line. The first point is 2 mm inferior to the oral commissure, injected very superficially, a second point was marked halfway along the tangential line, and a third point was marked in between the first two points. The authors recommend using a higher dilution ratio to administer the units more precisely in the upper half of the DAO. The average amount of toxin used per each DAO muscle is approximately 3–4 U.

**Ultrasound imaging of the depressor anguli oris muscle.**

**Figure 4:**
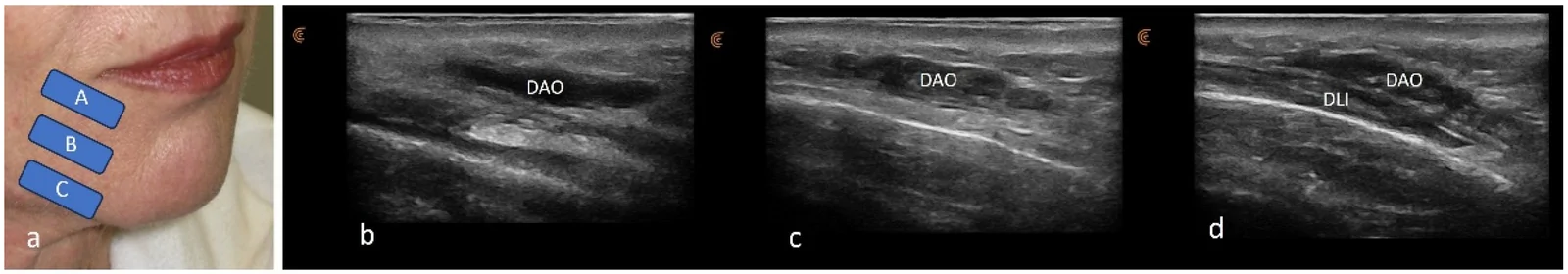
Different ultrasound probe positions used in the DAO scan. The blue rectangles represent the different positions of the probe used in the DAO scan. Position “A” scans the upper part of DAO, position “B” scans the mid part of DAO and position “C” scans the lower part of DAO (**a**). The DAO appears as a well-defined hypoechoic area located beneath the subcutaneous fatty tissue (**b**). The width of the DAO increases from its insertion to its origin, confirming its triangular shape. Its depth is lower at the medial margin and higher at the lateral margin, along the entire sagittal extension of the muscle (**c**). DAO partially overlaps the DLI, but unlike the DAO, which is simple to visualize, the DLI is more difficult to assess. Usually, DLI is easily visible, as a hypoechoic area, close to its bone origin, under the DAO (**d**).

The ultrasonographic imaging of the DAO provides detailed information about its morphology, location, and dimensions, increasing the safety and effectiveness of botulinum neurotoxin (BoNT) injections [42]. The DAO, as seen by ultrasound scanning, appears as a well-defined hypoechoic area located under the subcutaneous fatty tissue, which partially overlaps the underlying DLI, also appearing as a hypoechoic band. It is simple to visualize sonographically, and the borders of the muscle were easy to identify. By placing an ultrasound probe perpendicular to the labiomandibular sulcus in the upper, intermediate and lower part of the DAO, it is possible to identify the different dimensions and depths, as well as the different relationships with other muscles (Figure 4).

On the contrary, the DLI muscle is generally more difficult to assess due to the lower thickness and its continuity with the platysma muscles [43]. The good ultrasound definition of the DAO allows an excellent 3D description of its anatomy and its relationships with other nearby anatomical structures and superficial landmarks. This can improve the accuracy of BoNT-A injection, which is performed based on precise anatomical references, identifying optimal injection sites and redefining the plethora of suggested injection locations [44]. The ultrasound study defines the depth of the muscle with good precision, which is lower (4.74–5.79 mm) at the medial margin and higher (8.02–8.76 mm) at the lateral margin, along the entire sagittal extension of the muscle. The thickness of the DAO decreases in a cranio-caudal direction from 2.93 mm near the modiolus to 2.05 mm near the mandibular margin. The width of the DAO increases from 11.7 mm at its insertion to 15.03 mm at its origin [42] confirming the already described triangular shape of this muscle. Ultrasound allows us to evaluate the anatomical relationship between the DAO and the labiomandibular fold (LMF), named also the marionette line, which is a fundamental superficial skin landmark for BoNT-A injection. The DAO muscle is located directly beneath the marionette line in 100% of the investigated cases by Alfertshofer et al. [44]. The projection of the marionette line onto the muscle separated the DAO muscle into medial and lateral portions. Close to the oral commissure the DAO is approximately 25% medial and 75% lateral to the LMF, at midpoint it is about 35% medial and 65% lateral to the LMF and, at inferior margin of the mandible the DAO is roughly 52% medial and 48% lateral to the LMF skin projection. Alfertshofer et al. [44] define the midpoint along the LMF as the safest point and it is considered easy to reproduce clinically because it relies on individual distances along the LMF. In this point is present a sufficient DAO muscle mass medial to the injection site, serving as a “safe zone” from the medially located depressor labii inferioris (DLI) muscle. Differently, Zhang et al. [42] describe the medial border of DAO lateral to the LMF in a low percentage of subjects at upper and intermediate part (7.3–4.9%) but never at lower part, meanwhile the medial border is medial or superimposes to NLF in a high percentage of subjects (92.7% upper part, 86.1% middle part, 100% lower part). These findings suggest that injection entry points would target this muscle with better accuracy if they are at the middle or lower part of DAO and along the LMF. Zhang et al. [42], based on the average distance between the medial border of the DAO and the LMF, indicates that injection entry points would target the DAO with better accuracy if they are at mid or lower point along the LMF. Close to the modiolus the DAO is smaller or no muscle fibers were observed here in nearly one-third of patients. The injection of BoNT-A in this area may reduce the effectiveness of targeting and increases the risk of affecting other muscles that attach near the modiolus, such as the risorius, zygomaticus major, orbicularis oris, DLI, or buccinator muscles [42,44].

**Anatomy of the platysma muscle.**

**Figure 5:**
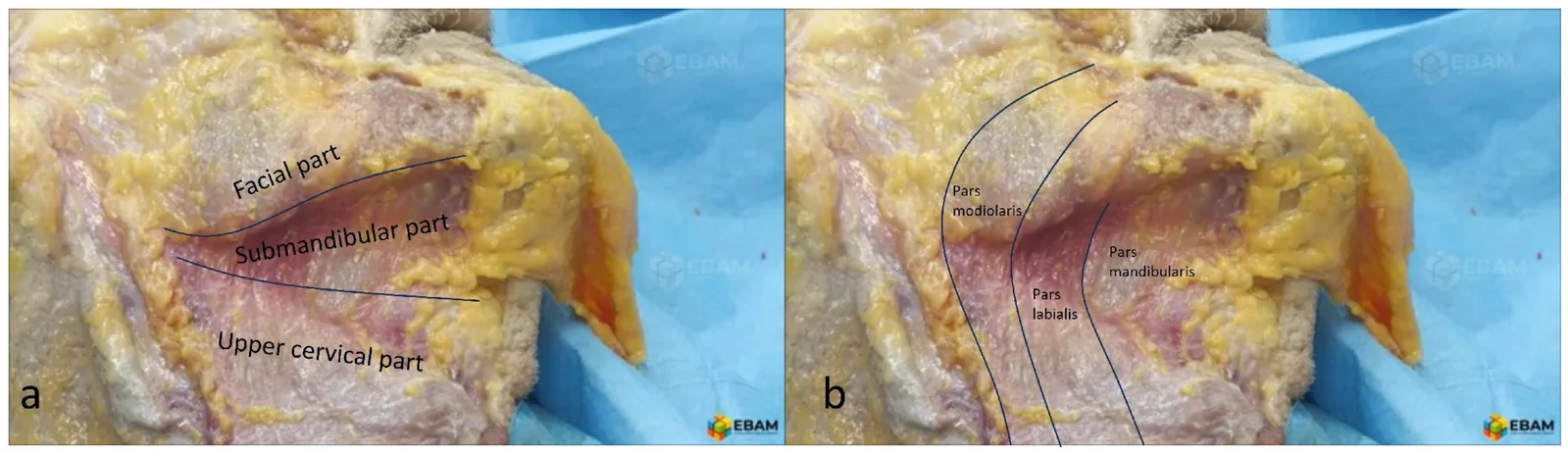
Anatomy of platysma muscle. (**a**) The topographic subdivision of the platysma muscle identifies a cervical part, a submandibular part and a facial part. (**b**) The subdivision by insertion patterns identifies the pars mandibularis (medial part), the pars labialis (intermediate part), and the pars modiolaris (lateral part).

The PL is a broad, flat, sheet-like, thin mimetic muscle located at the anterior neck. Its origin is the superficial fascia over the deltoid, trapezius, and pectoral muscles, and the clavicular region. The muscular fibres run upwards toward the lower face, cross over the sternocleidomastoid muscle and the submandibular gland. Reaching the cervicomental angle, the platysmal fibers make a turn of direction from vertical in the neck to more horizontal in the submandibular area and lower face (Figure 5) [45].

Along the upper region of the middle neck the platysmal fibers from the left and right sides decussate and interdigitate in a variable manner, but in the lower part of middle neck, the fibers are absent [46,47,48]. Two theoretical subdivision systems of the PL have been described: one is based on topographic location, the other on the insertion pattern. The topographic subdivision identifies a cervical part below the hyoid bone, a submandibular part between the mandible and the hyoid bone and a facial part above the mandible (Figure 5a). Subdivision by insertion patterns identifies the pars mandibularis, at the anterior third of the muscle, which attaches directly to the mandible. The intermediate part is named pars labialis; its fibers cross the body of the mandible, run under the DAO muscle, and insert directly into the lower lip dermis and intermingle with the orbicularis oris fibers. The more posterior third of the PL is the pars modiolaris, and its fibers insert into the buccinator and modiolus (Figure 5b) [49,50]. The PL muscle’s main functions are to pull the lower lip and the corners of the mouth downwards and outwards [51,52], to open the mouth by lowering the lower jaw [53], and to pull down the skin and soft tissues of the lower face and jawline.

The PL facial part receives motor innervation from the marginal mandibular nerve and sporadically from buccal branches of the cervical branch of the facial nerve. The submandibular segment and the upper portion of the cervical part are primarily innervated by the cervical branch of the facial nerve. The lower part of the cervical PL is innervated predominantly by sensory nerves, including the transverse cervical nerve, the great auricular nerve, and the supraclavicular nerve [45]. These are sensory nerves and don’t play a significant role in muscle contraction. This innervation pattern determines a cluster of motor endplates in the upper part of the PL muscle (facial, submandibular, and upper cervical segments), whereas the lower cervical portion of the PL muscle has mainly sensory innervation and a few motor endplates [54]. Yi et al., based on this motor endplates distribution, suggest that the injections of BoNT-A should be concentrated in the upper half of the PL to optimize the efficacy of the treatment. Injections into the lower PL, where there is mainly a sensory innervation, do not bring any additional benefit and may only increase the risk of complications.

**Injection techniques of the platysma muscle.**

Brandt and Bellman [55] described the PL treatment with BoNT-A in 1998. The authors proposed, after grasping the individual band and holding it firmly, the injection of BoNT-A in points at 1.0- to 1.5-cm intervals along the platysmal bands from the jaw-line to the lower neck. The units injected per treatment varied approximately from 50 to 100 U, depending upon the category of age-related neck degeneration. Kane [56] on 1999 published a similar technique injecting up to 20 U in larger bands or 5 U in smaller thinner bands. Other authors [57] reported a good improvement of platysmal bands injecting 15 U of Bont-A divided into six injections per band on each side of the neck. Afterwards, Levy [58] extended the treatment to the jawline with the so-called “Nefertiti lift” technique, injecting along and under each mandible and the upper part of the posterior platysmal band for a total of 15–20 U per side. The author reported that a total of 126 patients achieved an immediate and visible release of the downward pulling of the platysma muscle and had a noticeable re-contouring and elevation of the skin at the jawline. The other authors [59] stated that Nefertiti’s standard technique doesn’t have success in at least half of the patients and modified the technique, extending the treatment to all platysmal bands, and suggesting the injection of a larger amount of units at the most active part of PL and an injection points distribution based on the platysma contraction assessment. The total dose injected per session varying from 10 to 70 U with a median dose of 36 U. de Almeida et al. [60] described that, to enhance the lower facial contour, relax horizontal lines below the mandibular border, and reduce vertical smile lines lateral to the oral commissures, intramuscular injection of BoNT-A was performed into the MT muscle and into two horizontal lines of superficial injections above and below the mandible. The total dose administered was 16 U of Bont-A per side. In 2015, Wu [61] proposed injecting hyper-diluted Bont-A into the lower face and neck through multiple injections into the dermis or the interface between the dermis and the superficial layer of facial muscles. 20–28 U per mL of BoNT-A solution is used to deliver 100–120 microdroplets on each side. The results reported with this technique include a sharper cervicomental angle and jawline, lifted jowls, an improvement of platysmal bands, and a PL that is more adherent to the underlying neck. Wu postulates this mechanism of action: the microdroplets diffuse into the dermis and the superficial layer of the muscle, weakening only the superficial muscle fibres. In this way, the deep fibres become predominant and lead to a tightening of the PL muscle. Furthermore, intradermal BoNT-A deactivates sweat and sebaceous glands, which results in a decrease in volume and thickness of the dermal layer, along with subtle contraction and tightening of the overlying skin. A comparison between the injections along platysmal bands and the microbotox technique demonstrated that the first method has a higher capacity to reduce the bands and the second method a greater efficacy in improving the soft-tissue ptosis of the lower face and neck [62].

**Ultrasound imaging of the platysma muscle.**

**Figure 6:**
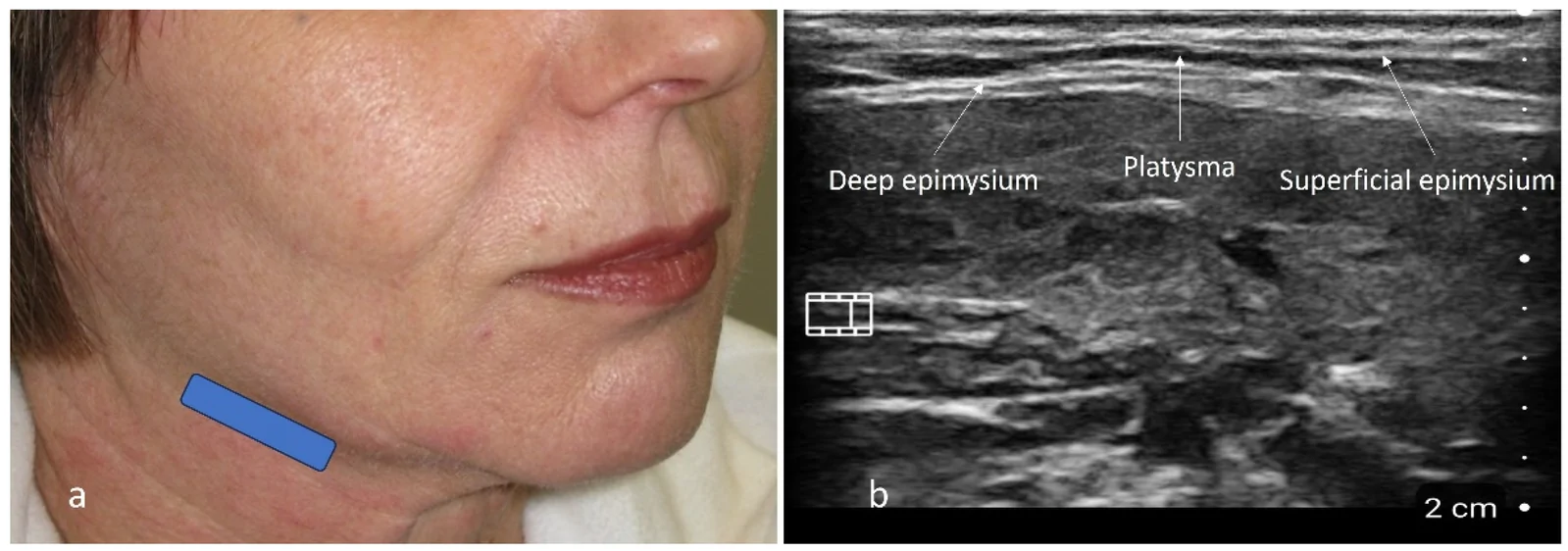
(**a**) The blue rectangle represents the position of the 20 MHz ultrasound probe. (**b**) By ultrasound, the platysma muscle is observed as a hypoechoic thin band under the thick hyperechoic epidermis, dermis, and subcutaneous tissue. The PL is enveloped by a superficial and deep epimysium, which appear as a hyperechoic line above and under the hypoechoic PL muscle.

By ultrasound, positioning the 20 MHz ultrasound probe parallel to and below the mandibular margin, the PL muscle is observed as a hypoechoic thin band under the thick hyperechoic epidermis, dermis, and subcutaneous tissue [63,64] (Figure 6). The PL is enveloped by a superficial and deep epimysium, which are connected by retinacula cutis superficiales of superficial fascia with the dermis, and by retinacula profunda of deep fascia with deeper neck structure [65]. The epimisium, being a fibrous structure, appears as a hyperechoic line above and under the hypoechoic PL muscle (Figure 6).

In the posterior cheek the PL muscle adheres to the fibrous parotid capsule, creating the posterior adherent zone, known as the platysma-auricular fascia. By ultrasound is possible to evaluate the PL presence and measure its thickness in all its extensions. Mean thickness varies from 0.48 mm to 0.67 mm at rest and increases especially along the medial and lateral bands during muscle contraction [66]. The possibility of identifying the anatomical depth of the PL by ultrasound enhances the capacity to inject the BoNT-A into the muscle thickness, increasing the treatment efficacy, allowing for a dosage reduction, and a safer treatment [66].

## 2. Results

**Results of the consensus—Mentalis muscle.**

The indications for mentalis (MT) muscle treatment with botulinum toxin that achieved strong consensus were correction of the “golf-ball chin” appearance (100%) and treatment of MT hyperactivity (92.8%). Chin projection enhancement and reduction of the horizontal labiomental fold were indicated by 42.8% and 28.5% of the panelists, respectively, without reaching consensus.

A combination of intradermal and intramuscular injections was used by 78.5% of experts (consensus), whereas intramuscular injection alone was used by only 21.4% (no consensus).

A certain degree of variability emerged regarding the injection points (IPs). No consensus was reached on this topic: 64.2% of panelists used two paramedian intramuscular IPs (one for each MT muscle), 21.4% used a single central IP, and 14.2% used three IPs (one central and two paramedian). Regarding intradermal injections, the panelists suggested 6–8 IPs for both MT muscles.

**Figure 7:**
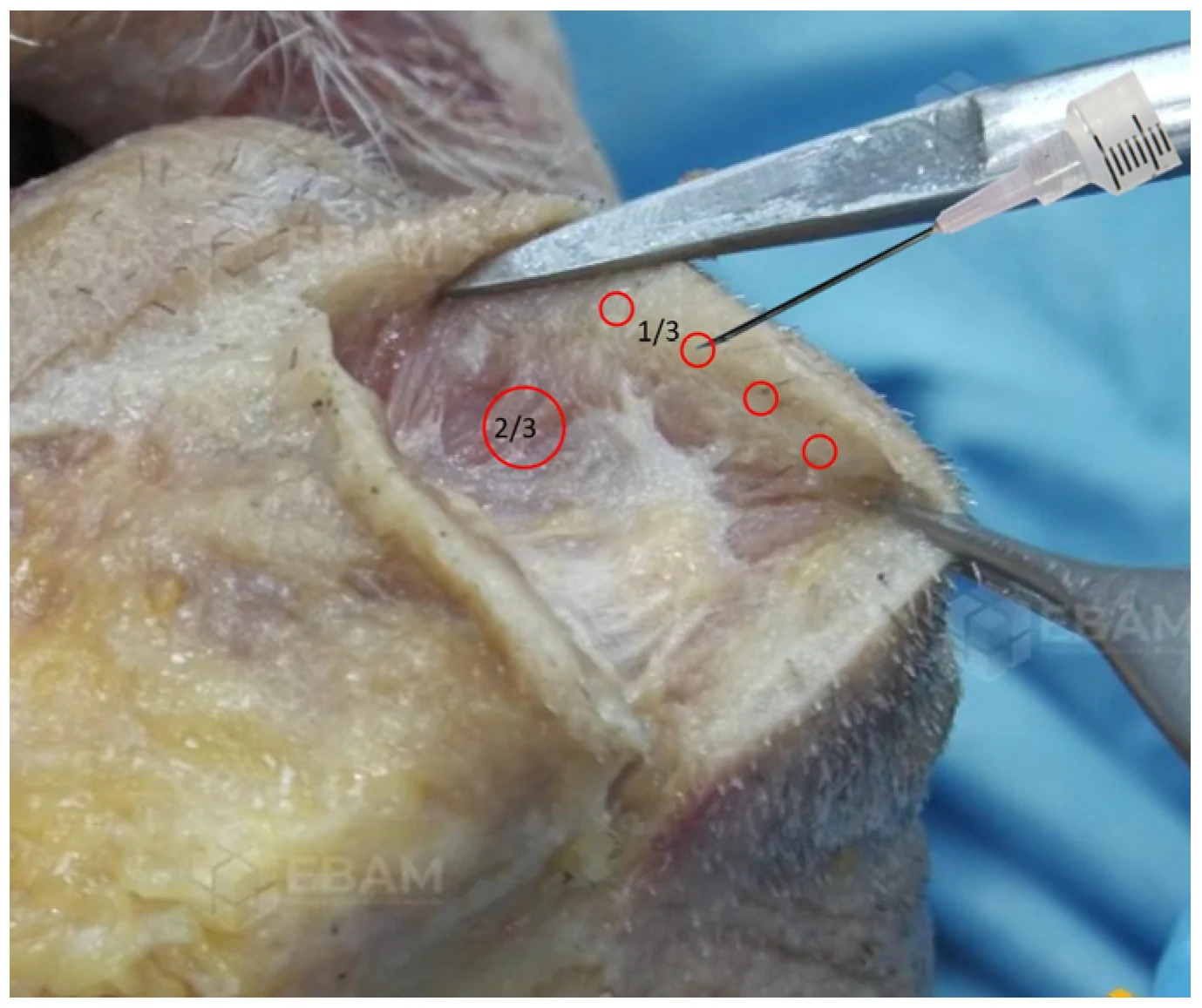
Treatment of the mentalis muscle. The dose of botulinum toxin injected into each MT muscle ranged from 1 to 6 U, with a mean dose of 3.9 U. Approximately two-thirds of the dose were administered intramuscularly (larger red circle) and one-third intradermally (smaller red circles).

The number of botulinum toxin units injected into each MT muscle ranged from 1 to 6 U, with a mean dose of 3.9 U. Approximately two-thirds of the total dose was administered intramuscularly and one-third intradermally (Figure 7).

The total dose for both muscles was approximately 8 U (Table 1).

**Results of the consensus—Depressor anguli oris muscle.**

The indication for DAO treatment with botulinum toxin that achieved strong consensus was correction of downturned mouth corners (100%). Reduction in DAO hyperactivity and lifting of the oral commissures each reached 85.7% agreement (consensus). Only 64.2% of panelists considered correction of marionette lines a valid indication, corresponding to a majoritarian agreement.

Intramuscular injection was used by 92.8% of experts, reaching strong consensus. In contrast, a combination of intramuscular and intradermal or subdermal injections was recommended by 58.3% of panelists (majoritarian agreement). Injection exclusively at the dermal or subdermal level did not reach consensus.

Overall, 85.7% of panelists used a single IP (consensus), whereas 35.7% used two IPs and only 7.1% used three IPs. The most frequently used injection site was the central portion of the DAO, midway between its bony origin and the modiolus (78.5%, consensus). Half of the panelists injected into the lower portion of the DAO, 35.7% injected into both the middle and lower portions, and 14.2% injected into the upper portion. Furthermore, 85.7% of panelists placed the IP lateral to the marionette line (consensus), whereas 28.5% injected along the marionette line and only 7.1% injected medial to it.

**Figure 8:**
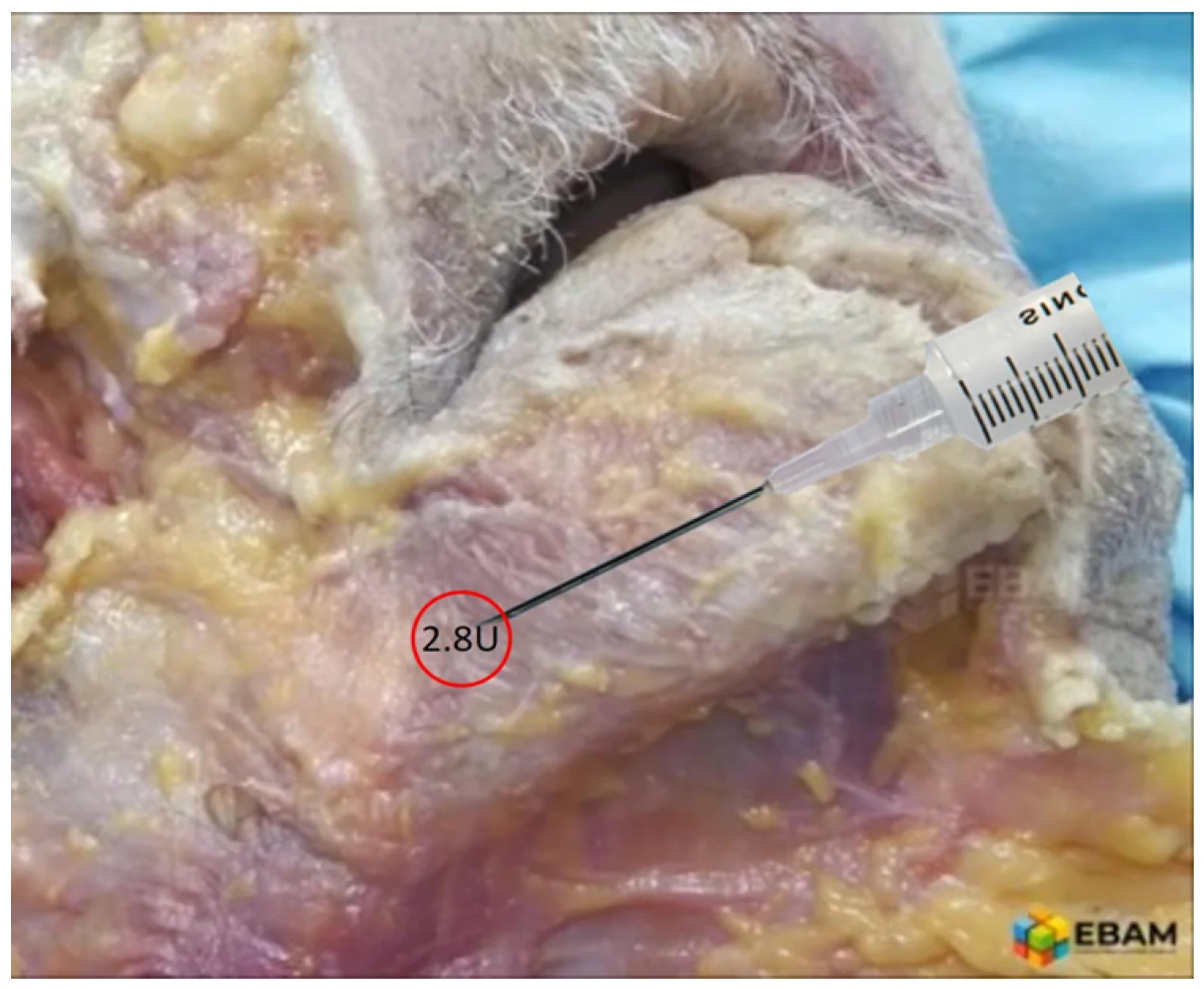
Treatment of the depressor anguli oris muscle. Most Italian panelists use a single injection point in the central portion of the DAO, midway between its bony origin and the modiolus, slightly lateral to the marionette line. The expert panel predominantly uses the intramuscular technique and recommends a mean dose of 2.8 U per side. Treatment of the depressor anguli oris muscle. Most Italian panelists use a single injection point in the central portion of the DAO (red circle), midway between its bony origin and the modiolus, slightly lateral to the marionette line. The expert panel predominantly uses the intramuscular technique and recommends a mean dose of 2.8 U per side.

The dose of botulinum toxin injected ranged from 1 to 5 U, with a mean dose of 2.8 U per DAO muscle (Figure 8, Table 1).

**Results of the consensus—Platysma muscle.**

The indication for platysma (PL) muscle treatment with botulinum toxin that achieved strong consensus was correction of platysmal bands (100%). Jawline recontouring (85.7%) and improvement in skin texture (78.5%) also reached consensus. Lifting of ptotic lowerface soft tissues reached 64.2%, corresponding to a majoritarian agreement.

**Figure 9:**
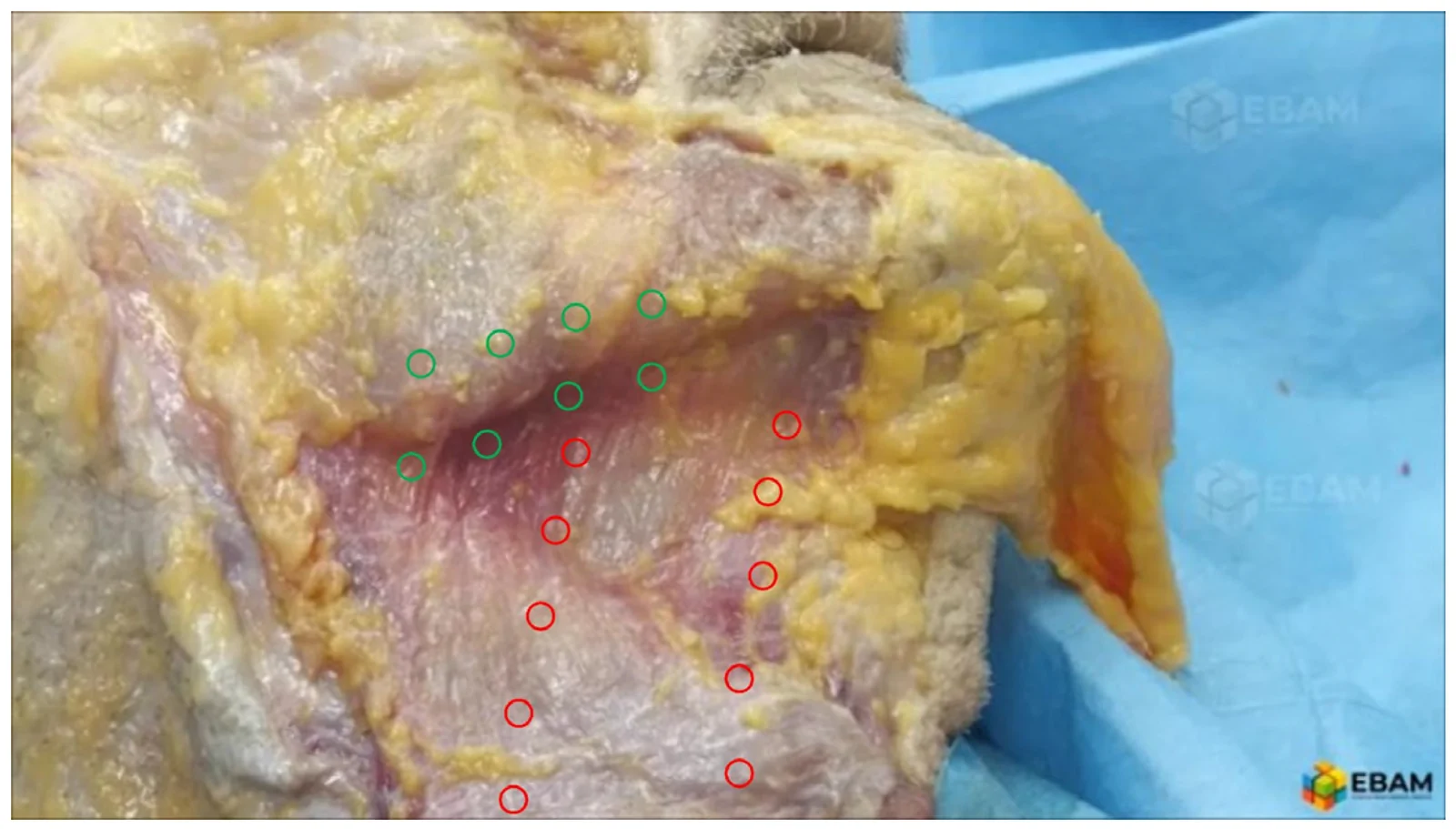
Treatment of the platysma muscle. Broad consensus was reached among the panelists regarding treatment of the platysma along the platysmal bands and mandibular border. The preferred injection planes were intramuscular and subdermal. The mean dose per side was 24.2 U. Treatment of the platysma muscle. Broad consensus was reached among the panelists regarding treatment of the platysma by injection points along the platysmal bands (red circles) and along the mandibular border (green circles). The preferred injection planes were intramuscular and subdermal. The mean dose per side was 24.2 U.

All experts combined different treatment approaches: 92.8% injected primarily along the platysmal bands (strong consensus), and 78.5% injected along the jawline (consensus) (Figure 9).

Only 57.1% of experts used the “microbotulinum” technique described by Woffles Wu, often with personal modifications (61).

Combined intramuscular and subdermal injections were used by 85.7% of experts, whereas intradermal injections were used less frequently (64.2%), reaching only majoritarian agreement. Overall, 85.7% of experts combined different injection planes.

Among the panel members, the dose administered per platysma muscle showed considerable variability, ranging from 9 to 50 U per side, with a mean dose of 24. U (Table 1).

Comparison with previous international consensus. A literature search was conducted using the MEDLINE and PubMed electronic databases for the period from January 2004 to December 2024. Additional studies were identified through manual screening of the reference lists of the retrieved articles. A total of 16 articles were included in the consensus review [14,17,18,66,67,68,69,70,71,72,73,74,75,76,77,78].

The recommended dosages for botulinum toxin treatment of the depressor anguli oris (DAO), mentalis (MT), and platysma (PL) muscles vary among published consensus statements, reflecting differences in patient populations, botulinum toxin formulations, and clinical objectives. Expert-opinion-based recommendations regarding botulinum toxin type and dosage are summarized in Table 2.

## 3. Discussion

The MT is the sole elevator of the lower lip and chin, providing essential vertical support. The EBAM-AITEB consensus identifies the “golf ball chin” and MT hyperactivity as the main indications for its treatment. The “golf ball chin” is the most widely recognized indication in the reviewed consensus, and MT hyperactivity also achieves strong consensus (over 92%). The deep mental crease, retrusion of chin projection, and lower lip eversion are indications that reached lower consensus levels. For MT treatment, most experts utilize either a single midline injection point or paired bilateral points, depending on the patient’s chin width and the severity of the hypertonia. Advanced techniques and specific patient presentations may require up to three, four, or five points [16,67,71,74]. Injection sites are typically located approximately 1 cm above the lower margin of the chin or jawline [70,72,74]. When a bilateral technique is used, the points are generally placed about 5 mm from the midline to prevent the toxin from spreading to the DLI. To avoid impairing oral functions such as speech or suction, injection points should be kept at a safe distance from the mouth; several consensuses recommend they be no closer than 1.5 cm from the lower lip [71,74,78]. One global guide suggests that a midline point can be supplemented by two additional lateral sites for enhanced correction [74]. Some practitioners employ a four-point technique, often using two deep injections at the base of the muscle and two superficial injections [77]. The most recent Asian consensus for letibotulinumtoxinA identifies three to five injection points as appropriate for correcting mentalis tension [78]. For standard treatment, deep intramuscular injections are common, often performed perpendicular to the skin [14,71,72,74]. Some practitioners angle the needle upward because the muscle originates cephalad to its dermal insertion site [67,78]. Some experts advocate superficial intradermal or subcutaneous injections directly into the dermal insertions of the muscle [75,77,78]. Good correspondence exists between international consensus and our consensus, although we suggest a higher dosage for treatment of both MT muscles (8 U vs. 6.5 U) (Table 3).

MT treatment with botulinum toxin has a relatively low incidence of complications. Lip asymmetry and ptosis may occur if the injection is placed too laterally or if the toxin diffuses into the DLI; the patient may then experience an asymmetrical smile or a drooping lower lip [68,70,71,74]. This can be managed by injecting 1–2 U of toxin into the opposite DLI to restore balance [16]. When the toxin spreads superiorly to involve the orbicularis oris, it can ause oral motor insufficiency, impairing essential lip functions. Patients may struggle with daily activities such as eating, drinking through a straw, or brushing their teeth [14,16,17,67,71]. A less common but specific complication is paradoxical bulging of the mentalis itself. This occurs when the muscle is only partially or unevenly relaxed, causing the untreated fibers to contract more prominently and create a distorted surface appearance [70].

The DAO pulls the corners of the mouth downward, contributing to a “gloomy” expression and the formation of marionette lines. The EBAM-AITEB (Italian) consensus reached varying levels of agreement regarding indications for DAO treatment with BoNT correction of downturned corners had strong consensus, while reduction of DAO hyperactivity and lifting of the mouth corner reached 85.7% consensus. Marionette line correction did not reach full consensus (64.2%). In previous consensus papers, the primary indications centered on correcting the downward-pulling forces that contribute to a “sad,” “sullen,” or “dissatisfied” facial expression [14,16,74]. Weakening DAO hyperactivity minimizes the downward pull on its dermal insertions, allowing the opposing zygomaticus muscles to lift the mouth corners back toward a neutral or upward position [14,16,18]. A secondary indication is the labio-mandibular fold (marionette lines). The DAO originates at the oral commissure and extends obliquely to the mandible; its hypertonia or overactivity leads to vertical marionette lines projecting downward from the corners of the mouth [14,18,20,22,72]. Chronic DAO hyperactivity can flatten the lips, resulting in a loss of perceived fullness, and may flatten the Cupid’s bow and diminish dental show during animation or smiling. DAO treatment can improve lip flattening and restore fullness [14,22].

Ultrasound imaging of the DAO muscle provides a detailed 3D description of its anatomy [42], and allows clinicians to determine the relationship between the DAO and the marionette line, which serves as a critical skin landmark. The DAO lies directly beneath the marionette line, and the projection of the marionette line divides the muscle into medial and lateral portions at different ratios along the muscle’s vertical extension. Ultrasound helps redefine safer injection locations to minimize side effects. The midpoint along the LMF is indicated as the safest injection site because this location has enough DAO muscle mass medial to the injection site to act as a “safe zone,” helping to avoid unintentional injection into the medially located DLI [42,44]. In contrast, a group of previous consensus papers agree to target the lower third of the DAO muscle, locating injection points at “1 cm from the jawline” or in the “lower portion of the DAO muscle” [14,16,17,74,78], whereas other consensus papers indicate a higher position along the midzone of the DAO muscle or closer to the corner of the mouth [68,70,72,75]. Usually, one injection point per side is suggested, but some authors indicate adding one additional point (totally two points per side). The injection depth for treating the DAO muscle varies between intramuscular [17,18,68,70,71,72] and superficial (subcutaneous/intradermal) [14,16,17,18,68,70,74,75,78]. Our expert panel reached consensus that placing one intramuscular injection point in the middle of the DAO and lateral to the marionette line helps target the muscle accurately while maintaining a “safe zone” to avoid unwanted neuromodulation of the medially located DLI muscle. This localization is consistent with the presence of a significant MEP cluster in the central portion of the DAO muscle, described by Lapatki et al. A complete correspondence of dosage exists between international consensus and our consensus: the mean total units per both sides indicated by the international consensus and our consensus are the same (5.6 U) (Table 3). Complications associated with botulinum toxin treatment of the DAO are primarily functional, occurring when the toxin spreads to adjacent muscles. The most frequent complication is asymmetrical smile, which occurs when the toxin diffuses medially into the DLI muscle [16,74,77]. If this asymmetry occurs, some experts recommend injecting 1–2 U of toxin into the opposite DLI to help restore smile balance [16,74]. If the toxin reaches the orbicularis oris, it can lead to impaired lip function, often described as a flaccid cheek or incompetent mouth [14, 15, 16]. This results in significant functional difficulties, such as drooling, problems with eating, drinking through a straw, suction, or speech impairments [14,67,71]. Some patients may have difficulty opening the mouth wide or experience a hanging mouth corner after treatment [70]. Among aesthetic complications are lip flattening and unsatisfactory facial expressions [14,78].

The PL muscle pulls the lower lip and the corners of the mouth downward and outward, assists in opening the mouth by lowering the lower jaw, and pulls down the skin and soft tissues of the lower face and jawline [51,52,53]. Among the expert panel of this consensus, the main indications for treating the PL muscle with botulinum toxin include correction of platysmal bands (100%), jawline re-contouring (85.7%), and skin texture improvement (78.5%). In contrast, lifting of ptotic lower face soft tissues reached only majoritarian agreement (64.2%). With ultrasound, the PL is observed as a thin, hypoechoic band located beneath the thick hyperechoic layers of the dermis and subcutaneous tissue. The usefulness of ultrasound evaluation in PL treatment appears less established than for MT and DAO, and further studies are needed. The higher presence of MEPs at the facial, submandibular, and upper cervical portions [45] of the platysma suggests that BoNT-A injections should be concentrated in the upper half of the PL to optimize treatment efficacy [54]. Several treatment aims have been described, ranging from targeted band reduction to lower face lifting, neck skin tightening, and attenuation of horizontal wrinkles. The wide variability of techniques is related to the variegated types of indications for the PL muscle, and these techniques are often used in combination to address platysmal bands, jawline definition, and skin texture. For platysmal band treatment, BoNT-A is injected along each band at 1.0 to 1.5 cm intervals, extending from the jawline down to the lower neck. The “Nefertiti lift” technique [58] focuses on the facial and submandibular segments of the platysma and the upper part of the posterior platysmal band. The “microbotulinum” technique, proposed by Wu, focuses on the superficial layers of the muscle and dermis by injecting hyper-diluted BoNT through multiple dermal or sub-dermal injections. The EBAM-AITEB panel reached strong consensus (92.8%) for injecting mainly along these bands and consensus (78.5%) for the “Nefertiti lift” technique; however, just over half of the experts (57.1%) utilize the “microbotulinum” technique. Injection depth varies among previous consensus papers. The IM injection level is the most popular depth for platysma treatment, particularly when addressing hypertonic bands; multiple consensus groups [18,67,68,71,72,74,75,77]), explicitly list intramuscular as the preferred or mandatory injection level for platysmal bands. Other consensuses identify both intramuscular and intracutaneous injection levels, and others suggest subcutaneous or intradermal levels, especially for lower face lift and neck skin tightening or when the “microbotulinum” technique is performed. Dosage variability for the PL muscle is high and spans a large range, even among the same consensus panel. The EBAM-AITEB consensus indicates a total mean dosage of 48.4 U per two sides, which is higher than the total mean dosage of all other consensuses (38.2 U per two sides) (Table 3). However, the dosage indicated by our expert panel is higher than the mean dosage of other consensus papers but consistent with dosages indicated in some of them [8,18,72]. The higher dosage proposed in this consensus may reflect increased panelist confidence in PL treatment based on published safety and efficacy data and clinical experience over the past 20 years. Dosage variability is demonstrated by the wide difference between the minimum dosage of 20 U proposed by the Canadian and US consensus (2004) [67] or the Asian consensus (2016) [73] versus the maximum dosage of 80 U proposed by another consensus (2023) [78]. An univocal orientation is not currently identifiable in the literature, and a shared dosage range has not yet been defined. A phase 3, multicenter, randomized, double-blind, placebo-controlled study [39] suggests 28 U per side in case of 1 band per side, 31 U in case of 1 band on one side and 2 bands on the other, and 36 U in case of 2 bands per side. These onabotulinumtoxin-A dosages are well tolerated, improve PL prominence severity, reduce signs of bother, and yield highly rated participant satisfaction. These dosages may serve as a benchmark for PL treatment, but variability in indications, injection techniques, and severity of PL hyperactivity makes it difficult to establish a shared dosage.

Information derived from ultrasound literature is useful for better defining the anatomical characteristics of muscles and consequently guiding injection techniques. Considering this information can improve treatment outcomes and safety. However, publications on this topic are still limited, and further studies are needed to improve knowledge and methods of use. Because facial muscles are thin, overlapping, and anatomically variable structures, good knowledge of facial muscle ultrasound imaging is necessary to allow ultrasound-based anatomical interpretation and to use ultrasound for injection guidance. Expert sonographers on the panel consider ultrasound useful for anatomical assessment of the MT and DAO muscles and, to a lesser extent, for the PL. Currently, insufficient data exist on the usefulness of ultrasound-guided injection of these muscles.

## 4. Conclusions

The demand for aesthetic treatment of the lower face and neck using BoNT-A continues to grow worldwide. This paper provides consensus-based recommendations on BoNT-A use for MT, DAO, and PL muscles, reviewing their anatomy, ultrasound imaging, published injection techniques, and comparing them with previous consensus papers. Standardized techniques, knowledge of anatomy, and ultrasound use for muscle assessment are key elements of optimal practice to maximize safety and effectiveness across potential uses.

## 5. Materials and Methods

An Italian consensus on MT, DAO, and PL treatment was performed following a three-round methodology. The first round consisted of constituting the KOL board, involving 1 scientific coordinator and 13 experts in the aesthetic use of botulinum toxin. All panelists are botulinum toxin injectors with at least 15 years of experience. The panel included 5 aesthetic doctors, 4 plastic and reconstructive surgeons, 3 cosmetic surgeons, 1 facial surgeon, and 1 facial sonographer. Six are experts in facial ultrasound, and seven are members of the board of directors of the AITEB, the Italian scientific society dedicated to aesthetic therapy with botulinum toxin; eight are members of the scientific committee of EBAM. The objects of the research statements were identified. During the second round, a digital survey (Evidence Based Aesthetic Medicine) was sent to the expert panel, and received answers were analyzed. Topics discussed in the survey for each muscle included: indications, injection depth, number of injection points, units injected in each point and muscle, percentage of treatment of each muscle, and treatment combination with the other two muscles object of the consensus. The third round consisted of a meeting during the 2025 annual EBAM Congress, during which an in-depth review of anatomy, injection techniques, and ultrasound imaging of each muscle was conducted, and the digital survey results were discussed and integrated with panel comments. At the end of the discussion, each panel member voted on the proposed recommendation. Levels of agreement were categorized as follows: >92% agreed = strong consensus; >70–95% agreed = consensus; >50–75% agreed = majoritarian agreement; ≤50% agreed = no consensus. The results were calculated as a percentage of completed forms for each muscle. To facilitate discussion, all emerging issues and final recommendations were summarized in a final document. This document was shared with all participants, who contributed a new version incorporating their final comments and suggestions. The final document was circulated, and the entire panel expressed agreement. The author reviewed consensuses published in America, Europe, and Asia over the last 20 years and compared data from these consensuses with data from this article. A literature search was conducted using MEDLINE and PubMed electronic databases for the period from January 2004 to December 2024. Manual search studies were also performed using the reference lists of articles included during the search process. Inclusion criteria were: literature limited to expert consensus; studies in English; including lower face treatment for aesthetic purposes; and describing dosage, number of injection points, and injection depth. The search included the following keywords used in different combinations: “botulinum toxin,” “consensus,” “aesthetic,” and “face.” The term “botulinum toxin AND consensus” retrieved 399 studies. The term “aesthetic, botulinum toxin” AND “consensus” retrieved 69 studies. The search term “face, botulinum toxin AND consensus” resulted in 69 studies. Thirty-one full-text articles were screened for eligibility, of which 15 were excluded due to lack of BoNT dosages and inappropriate paper type. A total of 16 articles were included in the consensus review. Generative artificial intelligence (GenAI) was used in this paper to extrapolate data from previous international consensus and compare them with those of our consensus.

## Figures and Tables

**Table 1:** Results of EBAM-AITEB consensus.

Muscle	Indications	Injection Level and Technique	Injection Points Per Side (Mean)	Dose Per Injection Point (Mean)	Total Dose Per Side (Mean)
Mentalis	Golf ball chin Mentalis hyperactivity	Intramuscular/Intradermal (combination of 1 im IP and 3–4 id IPs)	1 IPs intramuscular 3–4 IPs intradermal	1–4 U (2.78 U) intramuscular 1–2 U (1.5 U) intradermal	1–6 U (4 U)
DAO	Downturned mouth DAO hyperactivity Mouth corner lift	Intramuscular	1 IP in the middle of DAO and lateral to labio-mandibular line	2–4 U (2.8 U)	2–4 U (2.8 U)
Platysma	Platysmal bands Jawline contouring Skin texture	Intramuscular/subdermal (combination of im and id IPs along the platysmal band and the jawline)	3–30 IPs (16.7)	Variable based on the number of IPs	9–50 U (24.2 U)

**Table 2:** Comparison with previous international consensus.

Consensus Paper	Unit Per Both MT (Mean Dose) (Type of BoNT-A) (Number of IPs)	Unit Per Both DAO (Mean Dose) (Type of BoNT-A) (Number of IPs)	Units Per Both PL (Mean Dose) (Type of BoNT-A) (Number of IPs)
Carruthers [67] Canadian and US consensus	2–6 U (4 U) for women, 2–8 U (5 U) for men (OnaBoNT) (1–2 IPs total)	--	10–30 U (20 U) for women 10–40 U (25 U) for men (OnaBoNT) (2–12 IPs/band)
Raspaldo [14] French Consensus	2–6 U (4 U) for women, 2–8 U (4.5 U) for men, (OnaBoNT) (2 IPs/side)	4–10 U (7 U) (OnaBoNT) (1 IP/side)	30–40 U (35 U) (OnaBoNT) (2–4 IPs/band)
Maas [68] United States consensus	3–10 U (6.5 U) (OnaBoNT), 9–25 U (17 U) (AboBoNT) (1–2 IPs Total)	2–10 U (6 U) (OnaBoNT), 6–25 U (17.5 U) (AboBoNT) (1 IP/side)	30 U (OnaBoNT), 75 U (AboBoNT)
Carruthers [69] Global Consensus	4–10 U (7 U) (Ona & IncoBoNT) 5–25 U (15 U) (AboBoNT) (1 IP total)	2–15 U (8.5 U) (Ona & IncoBoNT), 5–20 U (12.5 U) (AboBoNT) (1 IP/side)	30–60 U (45 U) (Ona & IncoBoNT) 30–120 U (75 U) (AboBoNT)
Imhof [70] German consensus	4–8 U (6 U) (OnaBoNT) 10–20 U (15 U AboBoNT) (2 IPs total)	4–8 U (6 U) (OnaBoNT) 10–20 U (15 U) (AboBoNT) (1 IP/side)	20–40 U (30 U)(OnaBoNT) 50–125 U (87.5 U) (AboBoNT) (2–4 IPs/band)
Ahn [71] Korean consensus	5–10 U (7.5 U) (Ona & LetiBoNT) (1–2 IPs total)	6–10 U (8 U) (Ona & LetiBoNT) (2 IPs)	24–32 U (28 U) (Ona & LetiBoNT) (2–4/band)
Lorenc [76] United States consensus	4–5 U (4.5 U) (Ona & IncoBoNT) (2 IPs total)	5 U (Ona & IncoBoNT) (1 IP/side)	--
Yutskovskaya [72] Russian consensus	2–8 U (5 U) (IncoBoNT) (2 IPs total)	5 U (Ona & IncoBoNT) (1 IP/side)	50–60 U (55 U) (IncoBoNT) (2–4 IPs/band, 3 IPs/jawline)
Wu [73] Asian Consensus	4–8 U (6 U) (OnaBoNT) (1–2 IPs)	4–6 U (5 U) (OnaBoNT) (1 IPs/side)	10–30 U (20 U) (OnaBoNT) (10–12 IPs/side)
Sundaram [17] Asian Consensus	2–16 U (9 U) (IncoBoNT) (2–4 IPs total)	4–6 U (5 U) (IncoBoNT) (1 IPs/side)	40 U (IncoBoNT) (3–20 IPs/side)
Sundaram [18] Global consensus	4–10 U (7 U) (OnaBoNT) (1–4 IPs/side)	4–6 U (5 U) (OnaBoNT) (1–2 IPs/side)	36 U (OnaBoNT) (9 U/band across four bands) (3–6 IPs/band)
de Maio [74] Global consensus	4–8 U (6 U) (OnaBoNT) (3 IPs total)	4–8 U (6 U) (OnaBoNT) (1 Ip/side)	24–48 U (36 U) (OnaBoNT) (3–4 IPs/band, 6 IPs/jawline)
Kapoor [75] Indian Consensus	6–8 U (7 U) (OnaBoNT) (1–2 IPs total)	4–6 U (5 U) (OnaBoNT) (1 IP/side)	36 U (9 U per band across four bands) (3–4 IPs/band)
Kaminer [73] United States consensus	2–10 U (6 U) (Ona & PraboBoNT), 5–12 U (8.5 U) (IncoBoNT), and 4–24 U (14 U) (AboBoNT) (1–4 IPs total)	2–6 U (4 U) (Ona & PraboBoNT), 2.5–8 U (5.25) (IncoBoNT) and 5–15 U (10 U) (AboBoNT) (1–2 IPs/side)	--
Signorini [16] Italian consensus	8–10 U (9 U) (OnaBoNT) (1–2 IPs total)	4–8 U (6 U) (OnaBoNT) (1 IPs/side)	32 U (8 U per band across four bands) (OnaBoNT) (4 IPs/band)
Liu [78] Asian consensus	4–10 U (7 U) (LetiBoNT) (3–5 IPs total)	0.5–1 U (0.75 U) (LetiBoNT) (1 IPs/side)	60–100 U (80 U) (LetiBoNT) (40–60 IPs/side)

**Table 3:** Comparison of dosages between the EBAM-AITEB consensus and previous international consensus.

Muscle	EBAM-AITEB Consensus. Mean U Per Both Sides	International Consensus Review. Mean U Per Both Sides
Mentalis	8 U	6.5 U
DAO	5.6 U	5.6 U
Platysma	48.4 U	38.2 U

## Data Availability

No new data were created or analyzed in this study. Data sharing is not applicable to this article.

## Abbreviations

The following abbreviations are used in this manuscript:

BoNT-A	Botulinum toxin type A
MT	Mentalis
DAO	Depressor anguli oris
PL	Platysma
OO	Orbicularis oris
DLI	Depressor labii inferioris
LMF	Labiomandibular fold
EBAM	Evidence Based Aesthetic Medicine
AITEB	Associazione Italiana Terapia Estetica Botulino
KOL	Key Opinion Leader
